# Natural Small Molecules Targeting Mitochondrial Quality Control for the Treatment of Metabolic Diseases: Mechanisms, Novel Formulations, and Translational Perspectives

**DOI:** 10.3390/ph19091402

**Published:** 2026-09-05

**Authors:** Chongyan Zhao, Yuan Gao, Ting Zhou, Lei Sun, Qingsong Qu

**Affiliations:** 1 School of Traditional Chinese Medicine, Beijing University of Chinese Medicine, Beijing 102400, China; 2 School of Chinese Materia Medica, Tianjin University of Traditional Chinese Medicine, Tianjin 301617, China; 3 College of Traditional Chinese Medicine, Baotou Medical College, Baotou 014040, China

**Keywords:** mitochondrial quality control, metabolic diseases, natural small molecules, mitochondria, mitochondrial targeting strategy

## Abstract

Metabolic diseases are jointly driven by insulin resistance, chronic inflammation, lipotoxicity, and disrupted organelle homeostasis arising from sustained nutrient overload. These disorders substantially increase the risk of cardiovascular, renal, and other multi-organ complications and have become a major global public health burden. Mitochondria are central organelles that integrate energy metabolism with stress signaling. They participate in fatty acid *β*-oxidation, the tricarboxylic acid cycle, and oxidative phosphorylation, while also regulating reactive oxygen species generation, mitochondrial DNA-related inflammatory signaling, calcium homeostasis, and cell death. Under chronic metabolic stress, the mitochondrial quality control (MQC) system shifts from adaptive repair toward decompensation, characterized by impaired mitochondrial biogenesis, abnormal mitochondrial dynamics, defective mitophagy, disrupted proteostasis and mitochondrial unfolded protein response, increased oxidative stress, and impaired metabolic reprogramming. Natural small molecules possess structural diversity and multi-target, multi-pathway regulatory properties. They can modulate multiple MQC processes and improve mitochondrial function and metabolic phenotypes in preclinical models. Novel formulations, structural optimization, and mitochondria-targeted delivery can further improve their solubility, bioavailability, tissue exposure, and subcellular localization, thereby enhancing therapeutic efficacy and translational potential. This review systematically summarizes the mechanisms of MQC dysregulation in metabolic diseases, the evidence supporting natural small-molecule interventions, and strategies for formulation and delivery optimization. Future studies should strengthen causal validation of MQC, quantify intramitochondrial drug exposure, and incorporate clinically relevant endpoints to facilitate the translation of natural small-molecule MQC modulators.

## 1. Introduction

Chronic nutrient overload progressively overwhelms the capacity of the body to accommodate excess energy, disrupting the coordinated balance among adipose tissue storage, hepatic metabolism, and skeletal muscle substrate utilization [1]. This maladaptation promotes the development of obesity, type 2 diabetes (T2D), metabolic dysfunction-associated steatotic liver disease (MASLD), and metabolic dysfunction-associated steatohepatitis (MASH) [2,3,4]. As these diseases progress, insulin resistance, ectopic lipid deposition, chronic low-grade inflammation, and oxidative stress reinforce one another, ultimately contributing to sustained injury in the cardiovascular, renal, and nervous systems [5]. Although current lifestyle and pharmacological interventions can improve body weight, glycemic control, lipid profiles, and selected inflammatory markers, their ability to reverse fundamental metabolic dysregulation, organelle damage, and progressive organ dysfunction remains limited [6,7]. Elucidating how nutrient overload evolves from early compensatory adaptation into cellular injury and organ dysfunction is therefore essential for identifying shared pathological targets and developing more effective therapeutic strategies.

Mitochondria are central organelles that coordinate cellular energy metabolism and stress responses, with essential roles in glucose and lipid oxidation, oxidative phosphorylation, and redox homeostasis [8,9]. Under sustained nutrient overload and metabolic stress, mitochondrial respiratory efficiency progressively declines, accompanied by reactive oxygen species (ROS) accumulation, inflammatory signaling, and cellular injury [10]. To limit this damage while maintaining energy supply, cells must continuously identify, repair, and eliminate dysfunctional mitochondria and replenish the mitochondrial pool with newly generated functional organelles [11]. Mitochondrial quality control (MQC) is the dynamic regulatory network that coordinates mitochondrial biogenesis, remodeling, repair, and clearance to preserve mitochondrial integrity, proteostasis, genome stability, and metabolic function. During early metabolic stress, cells enhance mitochondrial biogenesis, adjust mitochondrial dynamics, and activate mitophagy and protein stress responses to repair or remove damaged mitochondria [12,13,14,15]. With persistent nutrient overload, however, mitochondrial repair and clearance mechanisms gradually become insufficient, leading to the accumulation of dysfunctional mitochondria, increased mitochondrial ROS (mtROS), impaired oxidative phosphorylation, and amplified metabolic inflammation. MQC dysfunction therefore represents a critical step linking nutrient overload to metabolic injury [16,17].

Natural small molecules have considerable potential for modulating MQC in metabolic diseases [18]. Their structural diversity and multitarget properties enable them to simultaneously influence energy sensing, oxidative stress, inflammatory signaling, autophagy, and lipid metabolism [19]. Studies have shown that several natural small molecules improve mitochondrial function and modulate MQC-related endpoints in preclinical models of obesity, T2D, MASLD, MASH, and associated complications [20,21,22,23]. However, their application is often limited by poor solubility, insufficient stability, low absorption, and inadequate tissue exposure in vivo, making it difficult to achieve therapeutically effective concentrations in diseased tissues and mitochondria [24]. Novel formulations, delivery systems, and structural optimization can improve systemic exposure, tissue distribution, and mitochondrial localization, thereby facilitating the translation of mitochondrial protective activity observed in vitro into therapeutic efficacy in vivo [25,26,27]. Integrating mechanistic studies with formulation and delivery optimization is therefore an important strategy for developing MQC-targeted therapies for metabolic diseases.

Accordingly, this review uses MQC as its central framework to summarize the mechanisms of MQC dysregulation in obesity, T2D, and MASLD/MASH, with particular emphasis on evidence that natural small molecules modulate mitochondrial biogenesis, dynamics, mitophagy, proteostasis, and redox homeostasis. We further review novel formulations, structural optimization approaches, and mitochondria-targeted delivery strategies and discuss the major challenges associated with mechanistic validation, drug exposure, safety, and clinical translation. This review aims to provide a framework for the discovery and development of natural small-molecule MQC modulators.

## 2. Mitochondrial Dysfunction and Quality Control Imbalance in Metabolic Diseases

### 2.1. Core Components of MQC and Their Roles in Metabolic Adaptation

**Figure 1:**
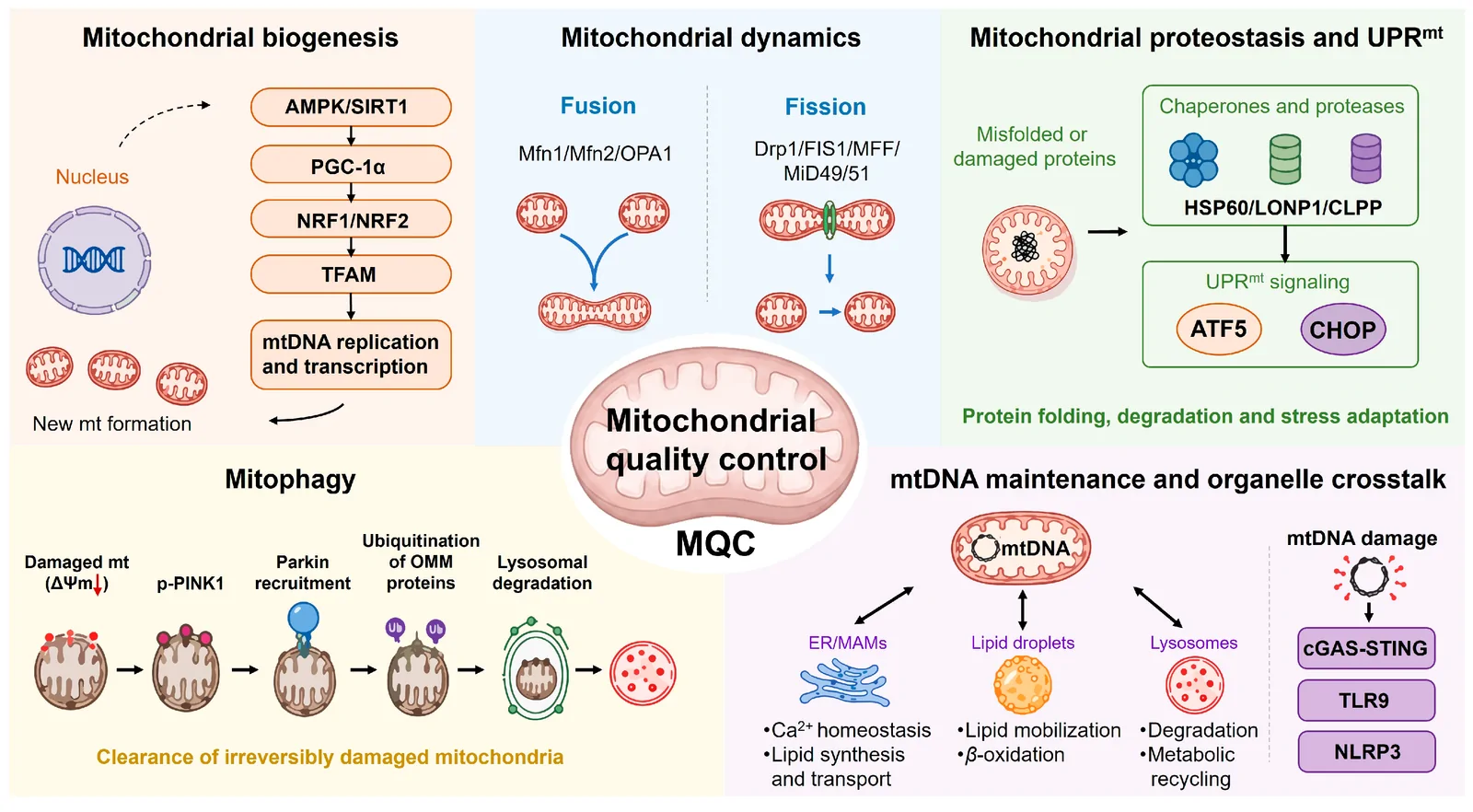
Major components of mitochondrial quality control. Mitochondrial quality control maintains mitochondrial homeostasis by coordinating mitochondrial biogenesis, fusion and fission, mitophagy, proteostasis and the mitochondrial UPR^mt^, mtDNA maintenance, and interorganelle communication. Together, these processes replenish functional mitochondria, segregate and eliminate damaged components, maintain protein and genome integrity, regulate metabolic exchange among organelles, and limit mtDNA-mediated inflammatory signaling.

MQC is a dynamic regulatory system that maintains mitochondrial structural and functional homeostasis (Figure 1). By coordinating mitochondrial biogenesis, fission and fusion, mitophagy, proteostasis, and mitochondrial DNA (mtDNA) maintenance, MQC facilitates the removal of damaged mitochondria and replenishment of the functional mitochondrial pool, thereby preserving energy metabolism and redox balance [28,29,30]. When MQC is disrupted, damaged mitochondria progressively accumulate, leading to insufficient ATP production, elevated mtROS, inflammatory activation, and cellular dysfunction, ultimately contributing to the progression of metabolic diseases [12,31,32].

#### 2.1.1. Mitochondrial Biogenesis

Mitochondrial biogenesis is essential for replenishing functional mitochondria and maintaining mitochondrial abundance and metabolic capacity [33]. During energy deprivation, exercise, or oxidative stress, an increased AMP/ATP ratio activates AMP-activated protein kinase (AMPK) and promotes the activity of the NAD^+^-dependent deacetylase sirtuin 1 (SIRT1) [34,35]. AMPK-mediated phosphorylation and SIRT1-mediated deacetylation cooperatively enhance the transcriptional coactivator activity of the peroxisome proliferator-activated receptor gamma coactivator 1-alpha (PGC-1*α*). PGC-1*α* subsequently coactivates nuclear respiratory factor 1 (NRF1) and GA-binding protein (GABP, also known as nuclear respiratory factor 2), thereby inducing the expression of mitochondrial transcription factor A (TFAM) and other nuclear-encoded mitochondrial proteins [36,37,38]. Following its import into mitochondria, TFAM regulates mtDNA replication, transcription, and stability [39,40]. This process coordinates the expression of nuclear- and mitochondrial-encoded proteins, facilitates respiratory-chain complex assembly, and restores oxidative phosphorylation capacity. The AMPK/SIRT1/PGC-1*α*/NRF1/2/TFAM axis therefore constitutes a central regulatory pathway for mitochondrial biogenesis [41,42].

#### 2.1.2. Mitochondrial Dynamics

Mitochondria continuously undergo fusion and fission to maintain the structural plasticity of the mitochondrial network and adapt to changes in energy demand, damage segregation, and cell division [41,43,44]. Outer mitochondrial membrane fusion is primarily mediated by mitofusin 1 (MFN1) and mitofusin 2 (MFN2), whereas inner membrane fusion depends on optic atrophy 1 (OPA1). Fusion enables adjacent mitochondria to exchange proteins, lipids, and mtDNA, thereby alleviating localized damage through functional complementation and preserving mitochondrial membrane potential and respiratory activity [45,46,47].

Mitochondrial fission is mainly mediated by dynamin-related protein 1 (DRP1). In response to metabolic stress or mitochondrial injury, cytosolic DRP1 is recruited to the outer mitochondrial membrane, where it interacts with receptors including Mitochondrial fission 1 protein (FIS1), Mitochondrial fission factor, and mitochondrial dynamics proteins. DRP1 then assembles into spiral complexes that constrict the mitochondrial membrane and complete fission [48]. Moderate fission segregates depolarized or structurally damaged mitochondrial fragments from the healthy network, facilitating their subsequent removal by mitophagy. Sustained DRP1 hyperactivation, however, causes excessive mitochondrial fragmentation, membrane depolarization, mtROS accumulation, and cell death [49,50]. The balance between mitochondrial fusion and fission therefore determines mitochondrial network integrity, damage segregation efficiency, and metabolic adaptability.

#### 2.1.3. Mitophagy

Mitophagy is the principal MQC mechanism responsible for eliminating irreversibly damaged mitochondria [51]. Under normal mitochondrial membrane potential, PTEN-induced kinase 1 (PINK1) is imported into the inner mitochondrial membrane, where it is continuously cleaved and degraded by mitochondrial proteases [52]. When the membrane potential declines, PINK1 import is impaired, resulting in its accumulation and autophosphorylation on the outer mitochondrial membrane. Activated PINK1 subsequently recruits and activates Parkin, which promotes the ubiquitination of outer mitochondrial membrane proteins. These ubiquitin chains are recognized by autophagy receptors such as OPTN, NDP52, and p62. Through their interaction with microtubule-associated protein 1 light chain 3 (LC3), these receptors promote the engulfment of damaged mitochondria by autophagosomes, which subsequently fuse with lysosomes to complete mitochondrial degradation [53,54,55].

In addition to the PINK1/Parkin pathway, mitochondrial outer membrane receptors can directly recruit autophagosomes through their LC3-interacting regions and mediate mitochondrial clearance under hypoxia, inflammation, and metabolic overload. Following outer mitochondrial membrane rupture, prohibitin 2 (PHB2) can function as an inner membrane mitophagy receptor by binding to LC3. Autophagy and beclin 1 regulator 1 (AMBRA1) also contribute to damage clearance by promoting autophagosome formation and mitochondrial recognition. After mitophagy, mitochondrial components are recycled, while coordinated mitochondrial biogenesis restores mitochondrial abundance and function [56,57,58,59].

Effective mitochondrial clearance requires the coordinated progression of damage recognition, autophagosome engulfment, and lysosomal degradation [60]. Changes in the expression of PINK1, Parkin, or LC3 reflect only individual stages of this process. Mitophagy should therefore be evaluated by assessing mitophagic flux, mitochondrial colocalization with autophagosomes or lysosomes, and the recovery of membrane potential, ATP production, and respiratory function [61].

#### 2.1.4. Mitochondrial Proteostasis and the Mitochondrial Unfolded Protein Response

Mitochondrial proteostasis depends on the coordinated import, folding, and degradation of proteins required for respiratory-chain assembly and metabolic enzyme activity [62]. When oxidative stress, impaired protein import, or respiratory-chain injury causes misfolded proteins to accumulate within mitochondria, cells activate the mitochondrial unfolded protein response (UPR^mt^) to alleviate proteotoxic stress and restore mitochondrial function [63,64].

During this response, molecular chaperones such as heat shock protein (HSP) 60 promote the refolding of abnormal proteins, whereas proteases including lon protease 1 and caseinolytic peptidase P (CLPP) remove proteins that cannot be repaired [65,66]. As the stress signal is transmitted to the nucleus, activating transcription factor 5 (ATF5) induces the expression of molecular chaperones, proteases, and antioxidant genes, while transcription factors such as C/EBP homologous protein (CHOP) also contribute to stress adaptation [67]. Moderate UPR^mt^ activation restores proteostasis and metabolic function. Persistent protein-folding stress that exceeds mitochondrial repair capacity may instead aggravate inflammation, metabolic dysfunction, and cell death [63].

#### 2.1.5. mtDNA Maintenance and Mitochondrial Interactions with Other Organelles

The integrity of mtDNA is essential for maintaining respiratory-chain function and oxidative phosphorylation [68]. Rather than being associated with conventional histone-based chromatin, mtDNA is compacted by TFAM and other proteins into mitochondrial nucleoids. Nevertheless, its localization within the redox-active mitochondrial environment and its dependence on specialized replication, repair, and turnover mechanisms make it susceptible to oxidative lesions and replication-associated errors [69,70]. Limited damage can be managed through DNA repair, selective degradation or turnover of damaged genomes, nucleoid remodeling, and compensatory replication. Persistent mtDNA damage or mutations impair mitochondrial gene expression and respiratory-chain assembly, ultimately compromising oxidative phosphorylation. Once released into the cytosol or extracellular space, mtDNA can function as a damage-associated molecular pattern. Cytosolic mtDNA primarily activates the cyclic GMP-AMP synthase–stimulator of interferon genes (cGAS–STING) pathway, whereas extracellular or endosomal mtDNA can engage toll-like receptor 9 (TLR9), and oxidized mtDNA can facilitate NLR family pyrin domain-containing 3 (NLRP3) inflammasome activation [71,72,73].

Mitochondria also form contact sites with the endoplasmic reticulum, lipid droplets, and lysosomes to coordinate substrate transport, energy metabolism, and damage clearance [74]. Mitochondria-associated endoplasmic reticulum membranes participate in lipid transport, Ca^2+^ exchange, mitochondrial fission, and autophagosome formation. Mitochondria-lipid droplet contacts facilitate the transfer of released fatty acids to mitochondrial *β*-oxidation, thereby limiting lipotoxicity [75,76]. Mitochondria–lysosome interactions contribute to damage recognition, mitochondrial network remodeling, and autophagic degradation [77]. Thus, mtDNA maintenance and interorganelle communication jointly connect mitochondrial structural integrity with metabolic adaptation and inflammatory responses.

### 2.2. MQC Dysregulation in Obesity, Insulin Resistance, and T2D

Obesity-associated MQC dysfunction exhibits marked tissue specificity and stage dependence [78]. During the early stages of energy surplus, adipocytes maintain metabolic adaptation by enhancing lipid storage, substrate oxidation, and mitochondrial biogenesis [79]. As adipocyte hypertrophy progresses, local hypoxia, endoplasmic reticulum stress, and inflammatory responses intensify, accompanied by impaired mitochondrial biogenesis, reduced oxidative phosphorylation efficiency, disrupted mitochondrial dynamics, and defective mitophagy. In brown and beige adipose tissues, reductions in mitochondrial abundance, uncoupling protein 1 (UCP1) dependent thermogenesis, and fatty acid oxidation further decrease energy expenditure [80,81]. mtROS and mtDNA released from damaged mitochondria activate the NLRP3 inflammasome and cGAS-STING pathway, promoting macrophage infiltration and lipolysis [72,73]. The resulting fatty acid flux to the liver and skeletal muscle contributes to systemic insulin resistance.

MQC abnormalities in skeletal muscle are major contributors to insulin resistance [82]. A mismatch between mitochondrial oxidative capacity and fatty acid supply in skeletal muscle leads to the accumulation of lipid intermediates, including diacylglycerols, ceramides, and acylcarnitines, which interfere with IRS/PI3K/AKT signaling and glucose transporter type 4 (GLUT4) translocation [83,84]. Impaired mitochondrial biogenesis, excessive fission, and defective mitophagy further increase mtROS generation and disrupt insulin signaling [85].

Pancreatic *β* cells are highly dependent on mitochondrial oxidative metabolism to generate ATP, trigger Ca^2+^ influx, and stimulate insulin secretion. Chronic glucotoxicity and lipotoxicity induce mitochondrial depolarization, mtROS accumulation, mtDNA damage, DRP1-mediated excessive fission, and defective mitophagy [86,87]. These alterations reduce ATP production and impair glucose sensing. Persistent stress further promotes *β*-cell apoptosis, senescence, and functional failure [88]. The progression from obesity to T2D can therefore be viewed as the progressive coupling of MQC dysfunction across adipose tissue, skeletal muscle, the liver, and pancreatic *β*-cells, ultimately forming a multi-organ pathological network linking lipotoxicity, inflammation, insulin resistance, and *β*-cell failure.

### 2.3. Mitochondrial Injury and Metabolic Inflammation in MASLD/MASH

The liver is a central regulator of lipid and glucose metabolism. During early MASLD, hepatocytes increase fatty acid *β*-oxidation, tricarboxylic acid cycle activity, and oxidative phosphorylation to accommodate substrate overload [89,90]. As fatty acid influx persists, mitochondrial oxidative capacity progressively becomes overwhelmed, resulting in electron leakage, elevated mtROS, NAD^+^/NADH imbalance, reduced ATP-production efficiency, and lipid peroxidation. These alterations promote the transition from hepatic steatosis to hepatocellular injury [91].

During disease progression, impaired mitochondrial biogenesis, DRP1-mediated excessive fission, defective MFN1/2- and OPA1-dependent fusion, and disruption of PINK1/Parkin-mediated mitophagy collectively promote the accumulation of dysfunctional mitochondria [92,93]. Concurrent lysosomal dysfunction, disrupted mitochondrial proteostasis, mtDNA damage, and abnormalities in mitochondria-associated endoplasmic reticulum membranes further impair Ca^2+^ homeostasis, lipid transport, and respiratory-chain function [94]. These changes establish a self-reinforcing cycle involving lipid overload, mitochondrial injury, and oxidative stress.

**Figure 2:**
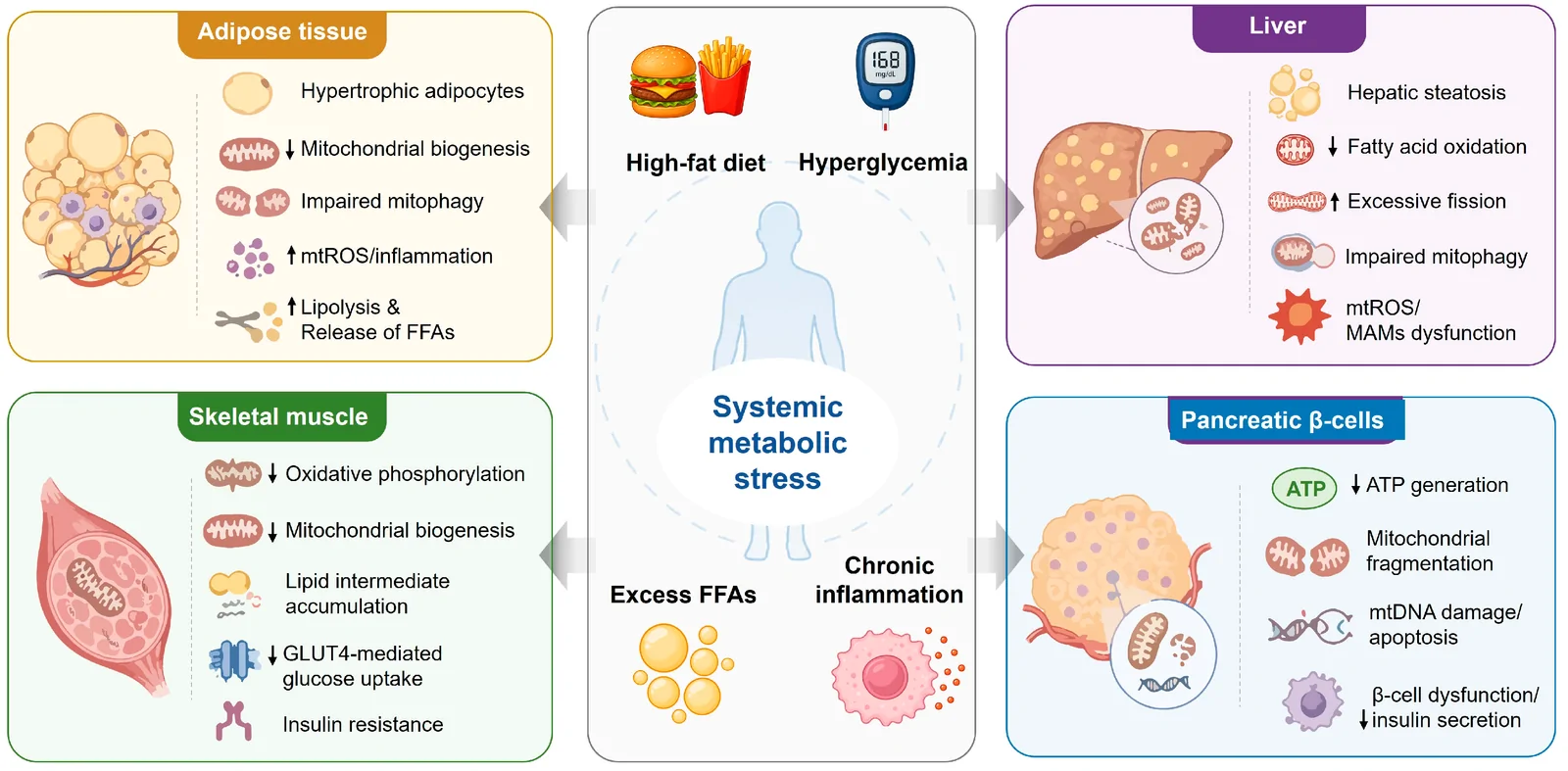
Tissue-specific mitochondrial quality control dysregulation during systemic metabolic stress. High-fat diet, hyperglycemia, excess free fatty acids, and chronic inflammation impair mitochondrial biogenesis, dynamics, mitophagy, and oxidative metabolism in adipose tissue, skeletal muscle, the liver, and pancreatic *β*-cells. These alterations promote adipocyte hypertrophy and lipolysis, insulin resistance, hepatic steatosis, and *β*-cell dysfunction, thereby reinforcing multi-organ metabolic deterioration. ↑ indicates an increase, and ↓ indicates a decrease.

Damaged mitochondria also activate cGAS-STING, TLR9, NF-κB, and the NLRP3 inflammasome through mtROS, oxidized mtDNA, cardiolipin, and lipid peroxidation products, thereby promoting inflammatory mediator release and Kupffer cell activation [95,96,97]. Hepatocyte apoptosis, pyroptosis, and ferroptosis further enhance immune-cell recruitment and stimulate hepatic stellate cell activation and extracellular matrix deposition, driving the progression of MASH and liver fibrosis [98,99]. Therapeutic strategies for MASLD/MASH should therefore restore coordination among mitochondrial biogenesis, dynamics, damage clearance, and energy metabolism to disrupt the mutually reinforcing cycle of lipid accumulation, metabolic inflammation, and fibrosis (Figure 2) [100].

## 3. Natural Small Molecules Targeting MQC in Metabolic Diseases

Natural small molecules are structurally diverse and often act on multiple targets and pathways, enabling them to attenuate metabolic injury by modulating several MQC-related processes, including mitochondrial biogenesis, dynamics, damage clearance, redox homeostasis, and interorganelle communication [19,101]. Current evidence can be broadly classified into three categories: mitochondrial biogenesis and metabolic adaptation, mitochondrial dynamics and mitophagy, and stress defense and homeostatic maintenance. Given the heterogeneity of disease models, experimental endpoints, and causal validation across studies, the effects of natural small molecules on MQC should be evaluated using integrated functional evidence, including mitochondrial respiration, membrane potential, morphology, and mitophagic flux [18,102].

### 3.1. Promotion of Mitochondrial Biogenesis and Metabolic Adaptation

The AMPK/SIRT1/PGC-1*α* axis is a major pathway through which natural small molecules promote mitochondrial biogenesis and metabolic adaptation [34]. AMPK enhances PGC-1*α* activity through direct phosphorylation and SIRT1-dependent deacetylation [103]. Activated PGC-1*α* subsequently induces mitochondrial biogenesis-related factors, including NRF1, GABP, and TFAM, thereby promoting mtDNA replication, respiratory chain assembly, and oxidative metabolism [104].

Luteolin activates AMPK/PGC-1α signaling in Western diet-fed mice, enhances mitochondrial biogenesis and mitochondrial enzyme activity, and reduces hepatic lipid accumulation [105]. In a high-fat diet-induced model of skeletal muscle insulin resistance, astaxanthin activates AMPK, promotes mitochondrial remodeling and biogenesis, and improves oxidative metabolism, glucose tolerance, and exercise capacity [106]. Melatonin similarly enhances mitochondrial biogenesis in skeletal muscle and the liver through pathways involving CaMKII, AMPK, and PGC-1*α*, thereby improving glucose and lipid metabolism in obese or diabetic animals [107,108]. Fucoxanthin binds to and inhibits PKM1, reducing glycolytic flux in skeletal muscle while enhancing PGC-1*α*/TFAM-mediated mitochondrial biogenesis and PPAR*α*/CPT1-dependent fatty acid *β*-oxidation. These effects reduce lipid accumulation and improve insulin resistance [109]. In human studies, short-term resveratrol supplementation produces calorie restriction-like metabolic effects and modulates AMPK, SIRT1, and PGC-1*α*-associated energy metabolism [110].

Saponins and quinones have shown particularly consistent effects on mitochondrial remodeling in adipose tissue. Ginsenoside F2 suppresses adipogenesis and promotes mitochondrial biogenesis through AMPK activation [111]. Ginsenoside Rg3 restores inflammation-impaired thermogenesis in brown and beige adipose tissues, accompanied by increased UCP1 expression, oxygen consumption, and fatty acid oxidation [112,113]. Tanshinone I activates AMPK, enhances fatty acid oxidation and mitochondrial biogenesis, and promotes brown adipose tissue activation and white adipose tissue browning, thereby attenuating high-fat diet-induced obesity [114]. Tanshinone IIA and its derivative TAN20 increase mitochondrial abundance and thermogenic activity through the AMPK/PGC-1*α*/UCP1 axis, improving obesity and insulin resistance in a UCP1-dependent manner [115,116].

In addition to promoting mitochondrial biogenesis, enhancing mitochondrial substrate oxidation is important for alleviating lipotoxicity. Baicalin directly targets hepatic CPT1A and facilitates the transport of long-chain fatty acids into mitochondria for *β*-oxidation, thereby ameliorating diet-induced obesity and hepatic steatosis [117]. Alnustone binds to the Ca^2+^ binding sites of calmodulin, increases cytosolic and mitochondrial Ca^2+^ levels, and enhances mitochondrial fatty acid *β*-oxidation. Consequently, it reduces hepatic steatosis and insulin resistance in MASLD mice and ameliorates MASH-associated liver fibrosis [118]. Berberine restores mitochondrial energy homeostasis through PGC-1*α* in diabetic nephropathy models, further suggesting that natural small molecules can protect metabolic organs by regulating mitochondrial biogenesis and substrate utilization [119].

### 3.2. Regulation of Mitochondrial Dynamics and Mitophagy

Mitochondrial fusion and fission maintain network integrity and facilitate the segregation of damaged mitochondrial components [43]. Persistent exposure to high glucose, fatty acids, or inflammatory stimuli can induce excessive DRP1 activation, resulting in mitochondrial fragmentation, membrane depolarization, mtROS accumulation, and cell death. Xu et al. [120] found that epigallocatechin gallate inhibited DRP1-associated mitochondrial apoptotic signaling and alleviated high-glucose-induced *β*-cell dysfunction, suggesting that it protects *β* cells by modulating the interplay between mitochondrial dynamics and cell death. In diabetic mice and high-glucose-treated endothelial progenitor cells, astragaloside IV decreases DRP1 expression while increasing MFN1, MFN2, and OPA1 expression. It also attenuates mtROS accumulation and mitochondrial dysfunction through the GSK-3*β*/Nrf2 axis [121]. Coenzyme Q10 preserves mitochondrial dynamics, promotes white adipose tissue browning, and improves obesity-related metabolic abnormalities in high-fat diet-fed animals [122].

Mitophagy is responsible for the selective removal of irreversibly damaged mitochondria and represents an important component of MQC targeted by natural small molecules. Quercetin activates AMPK-dependent hepatic mitophagy, reducing lipid accumulation and mitochondrial injury and thereby improving NAFLD-related phenotypes [21]. Quercetin can also promote fatty acid *β*-oxidation by stimulating lipophagy; however, because lipophagy primarily reflects lipid droplet mobilization, it should be evaluated separately from mitophagy [123]. In palmitate-treated HepG2 cells, hesperetin restores mitochondrial membrane potential, ATP production, and fatty acid oxidation while enhancing PINK1/Parkin-dependent mitophagy and suppressing NLRP3 inflammasome activation and cell death [124].

Lycopene downregulates DRP1, increases MFN2 and OPA1 expression, and activates PINK1/Parkin-mediated mitophagy. These effects restore mitochondrial membrane potential and ATP production, reduce oxidative stress, hepatocyte apoptosis, and hepatic lipid accumulation, and ultimately ameliorate MASLD and preserve liver function [125]. Myricetin stabilizes PINK1 and activates PINK1/Parkin-dependent mitophagy, thereby restoring mitochondrial function and fatty acid *β*-oxidation. It consequently reduces hepatic steatosis, inflammation, fibrosis, and insulin resistance in MASLD/MASH models [126].

Kaempferol improves mitochondrial homeostasis in diabetic nephropathy by coordinating mitochondrial dynamics and mitophagy. In diabetic nephropathy rats and advanced glycation end product-treated HK-2 cells, kaempferol increases MFN1 and OPA1 expression, decreases DRP1 and FIS1 expression, and enhances PINK1/Parkin-mediated mitophagy. These changes restore mitochondrial membrane potential and ATP production while reducing oxidative stress, inflammation, and apoptosis [127]. Diosgenin restores autophagy through the CaMKK2/AMPK/mTOR pathway and activates PINK1/MFN2/Parkin-mediated mitophagy. It also suppresses excessive mitochondrial fission and promotes fusion by downregulating DRP1 and phosphorylated DRP1 while increasing MFN1, MFN2, and OPA1 expression, thereby alleviating renal injury in type 2 diabetic nephropathy [128].

### 3.3. Improvement of Mitochondrial Redox Homeostasis, Interorganelle Communication, and Cell Death Signaling

Metabolic overload increases electron leakage from the respiratory chain and mtROS production, leading to oxidative damage to proteins, membrane lipids, and mtDNA [129]. Natural small molecules frequently alleviate these effects by regulating SIRT3, Nrf2, and mitochondrial antioxidant systems. Nevertheless, general antioxidant effects should be distinguished from direct MQC modulation. A more convincing MQC effect requires that decreased mtROS be accompanied by improvements in mitochondrial respiration, membrane potential, dynamics, mitophagy, or damage clearance [29].

Curcumin is one of the most extensively studied polyphenols in this field. It alleviates hepatic steatosis by improving mitochondrial function through SIRT3 in overfed rats and steatotic cells [20]. Kuo et al. further demonstrated that curcumin reduced hepatic NF-κB activity, thiobarbituric acid-reactive substances, and inflammatory cytokine expression while increasing glutathione levels in ob/ob mice. Curcumin also restored mtDNA copy number, NRF1 and TFAM expression, mitochondrial complex I activity, and ATP production and reduced hepatic lipid accumulation [130]. These findings indicate that curcumin alleviates obesity-associated hepatic steatosis by improving redox homeostasis, promoting mitochondrial biogenesis, and restoring respiratory chain function.

In NASH mice and palmitate-treated AML12 cells, nobiletin improves mitochondrial complex I activity, ATP production, mitochondrial Ca^2+^ uptake, and ROS homeostasis while reducing hepatocyte death, inflammation, and fibrosis [22]. By linking respiratory chain function and Ca^2+^ homeostasis to disease phenotypes, this study identifies mitochondrial Ca^2+^ regulation as a potential mechanism through which natural small molecules may ameliorate MASH. Matrine alleviates lipotoxicity-induced endoplasmic reticulum stress and mitochondrial injury by regulating SERCA-mediated Ca^2+^ homeostasis. In animal and cellular models, it suppresses PERK-, IRE1-, and ATF6-related signaling, restores mitochondrial membrane potential, and reduces ROS production, apoptosis, and hepatic lipid accumulation [131].

mtROS accumulation and mtDNA damage also connect mitochondrial dysfunction with metabolic inflammation. Nrf2 activation enhances antioxidant and detoxification systems and reduces lipid peroxidation and mitochondrial injury [132]. Restoration of mitochondrial function may further suppress inflammatory pathways. Berberine modulates AMPK, Nrf2, NF-*κ*B, and NLRP3-related metabolic and inflammatory signaling while improving glucose metabolism and insulin sensitivity [133,134].

Mitochondrial proteostasis and the UPR^mt^ are important components of MQC. These processes regulate the folding, import, degradation, and assembly of respiratory chain subunits, whereas their disruption impairs electron transport and aggravates oxidative injury [135,136]. Nicotinamide riboside induces a SIRT1/SIRT3-dependent UPR^mt^ and increases CLPP and HSP10 expression, thereby promoting respiratory chain assembly, fatty acid *β*-oxidation, and energy metabolism. These effects prevent and reverse high-fat, high-sucrose diet-induced hepatic steatosis [137].

The iridoid glycoside agnuside directly binds to and stabilizes the complex I assembly factor NDUFAF6, reducing its ubiquitination and degradation. Agnuside consequently promotes the assembly of complex I and respiratory chain supercomplexes, enhances mitochondrial respiration, ATP production, and adipose tissue thermogenesis, and improves high-fat diet-induced obesity and insulin resistance [138]. However, evidence that natural small molecules directly regulate core UPR^mt^ components in metabolic diseases remains limited. Most studies have instead focused on endoplasmic reticulum stress, JNK/CHOP signaling, and mitochondrial apoptotic pathways. For example, dihydroartemisinin alleviates metabolic injury by regulating SERCA-dependent Ca^2+^ homeostasis and endoplasmic reticulum stress-associated mitochondrial death signaling, respectively [139,140].

Overall, natural small molecules can restore MQC through multiple mechanisms, including the promotion of mitochondrial biogenesis, coordination of mitochondrial dynamics and mitophagy, improvement of redox homeostasis, and maintenance of mitochondrial proteostasis (Table 1). Future studies should incorporate mitochondrial respiratory measurements, mitophagic flux assays, target-specific interventions, and genetic validation to establish their direct effects on the MQC network and clarify their translational potential.

## 4. Formulation, Structural Optimization, and Mitochondria-Targeted Delivery

Although many natural small molecules can improve mitochondrial function, their therapeutic development is often limited by poor water solubility, insufficient stability, low gastrointestinal absorption, and rapid metabolism. These unfavorable drug-like properties may prevent effective concentrations from being achieved in target tissues and mitochondria. When the concentrations required for in vitro activity substantially exceed the achievable free-drug exposure in vivo, the associated MQC effects are unlikely to translate into consistent therapeutic benefits [19]. Therefore, formulation development, structural optimization, and targeted delivery are essential for translating natural small molecules into viable therapeutics.

Current strategies can be organized into three sequential levels. First, formulation approaches, including liposomes, nanoemulsions, and polymeric nanoparticles, are used to improve aqueous solubility, chemical and metabolic stability, gastrointestinal absorption, and systemic exposure [141,142]. Second, tissue-directed delivery systems, such as liver-targeted liposomes, locally administered hydrogels, and inflammation-responsive carriers, aim to increase drug retention and exposure at diseased sites while limiting off-target distribution [143,144]. Third, subcellular targeting strategies employ membrane-potential-driven lipophilic cations, particularly triphenylphosphonium (TPP^+^), mitochondria-penetrating peptides, fusogenic MITO-Porter systems, and mitochondria-targeted or stimuli-activated prodrugs to enhance mitochondrial accumulation and/or organelle-selective drug release [145]. Together, these approaches establish a stepwise optimization framework that progresses from improving drug-like properties to enhancing tissue exposure and ultimately achieving greater mitochondrial delivery specificity (Table 2).

### 4.1. Nanocarriers and Tissue-Targeted Delivery

Liposomes, polymeric nanoparticles, and other nanocarriers can improve the dispersibility and stability of hydrophobic natural small molecules, prolong systemic circulation, and increase drug exposure in metabolically active organs such as the liver. The high-phosphatidylcholine curcumin liposome hPLipo/Cur, composed of DSPC, cholesterol, and DSPE-PEG2000, reduced hepatic lipid accumulation and macrophage infiltration more effectively than free curcumin in experimental steatohepatitis. Mechanistically, hPLipo/Cur inhibited NRF2 degradation and promoted its nuclear translocation, thereby reducing mtROS production, lipid peroxidation, and ferroptosis. These findings suggest that enhanced solubility and tissue delivery can amplify the effects of curcumin on mitochondrial oxidative stress [146].

A galactose-modified oxidized starch–lysozyme nanocarrier enhances resveratrol uptake by targeting the asialoglycoprotein receptor expressed on hepatocytes. In a high-fat diet-induced fatty liver disease model, this formulation reduced hepatic lipid accumulation and insulin resistance and modulated AMPK/SIRT1 related metabolic signaling. Compared with free resveratrol, enhanced liver-selective uptake enabled the formulation to improve hepatocellular energy metabolism and lipid homeostasis more effectively [147].

Similarly, galactose-modified PLGA-PEG nanoparticles improved the aqueous solubility, sustained release, and hepatocyte uptake of epicatechin. In high-fat diet-induced MASLD mice, this formulation reduced hepatic lipid accumulation, improved insulin sensitivity, activated AMPK, inhibited mTORC1, and restored autophagic activity. These effects were accompanied by increased mitochondrial membrane potential, reduced mtROS production, and restored expression of mitochondrial biogenesis-related proteins [148].

Adipose tissue-targeted nanocarriers may enhance mitochondrial thermogenesis. Tian et al. developed AE@PEP-Lip, an SS-31-functionalized cationic liposome co-delivering allicin and empagliflozin. The formulation accumulated in white adipose tissue and mitochondria, reduced mtROS and mitochondrial fragmentation, and enhanced mitochondrial respiration and thermogenesis. Allicin also activated AMPK signaling and increased uncoupling protein expression. AE@PEP-Lip promoted white adipose tissue browning in obese mice and reduced localized fat deposition in a porcine model, demonstrating the potential of combined tissue- and mitochondria-targeted delivery to improve metabolic efficacy [149].

### 4.2. Mitochondria-Targeted Delivery Strategies

Mitochondria-targeted delivery aims to increase the effective concentrations of natural small molecules within the mitochondrial matrix or near the inner or outer mitochondrial membrane, enabling direct modulation of the respiratory chain, redox systems, and MQC-related proteins [150]. TPP conjugation is one of the most widely used mitochondrial targeting strategies. Driven by the negative potential across the inner mitochondrial membrane, TPP-containing compounds preferentially accumulate within mitochondria. TPP is therefore frequently conjugated to natural small molecules, lipids, polymers, or prodrugs [145].

Che et al. conjugated galactose to whey protein isolate through the Maillard reaction to target hepatocyte asialoglycoprotein receptors and further introduced TPP to construct the AST@TPP-WPI-Gal astaxanthin nanocarrier. This formulation accumulated within mitochondria in steatotic HepG2 cells and enhanced the antioxidant and antilipogenic effects of astaxanthin. In high-fat diet-induced NAFLD mice, it improved serum lipid profiles and liver function and reduced hepatic lipid accumulation by approximately 40% more than free astaxanthin [151].

Mitochondria-targeted micelles can also increase the subcellular exposure of curcumin. Zhang et al. [152] developed self-assembled Cur-CTPP-PEG-PCL micelles with an encapsulation efficiency of 92.88%. These micelles exhibited efficient endosomal escape and mitochondrial colocalization. Compared with free curcumin and non-targeted micelles, Cur-CTPP-PEG-PCL prolonged the in vivo retention of curcumin and increased its bioavailability. In mice with CCl_4_-induced liver fibrosis, the formulation produced greater reductions in liver enzyme levels and collagen deposition.

The efficiency of mitochondria-targeted delivery depends on mitochondrial membrane potential, intracellular trafficking, and carrier safety. The uptake of lipophilic cations such as TPP relies on mitochondrial membrane potential. Therefore, severe mitochondrial damage or depolarization may reduce carrier accumulation. Excessive cationic accumulation may also disrupt the respiratory chain, membrane potential, and membrane permeability [153]. Moreover, nanocarriers may become trapped within endosomes or lysosomes after cellular uptake, limiting their transport to mitochondria. Mitochondrial exposure should therefore be confirmed using subcellular fractionation followed by quantitative drug analysis, confocal colocalization, mass spectrometry imaging, or radiotracer techniques [154]. These measurements should be integrated with assessments of mitochondrial respiration, membrane potential, and MQC-related functions to determine the biological effects of the delivery system.

### 4.3. Structural Optimization and Translational Evaluation

Structural optimization can alter the solubility, metabolic stability, membrane permeability, and subcellular distribution of natural small molecules through scaffold functionalization, linker modification, and conjugation with targeting moieties [19]. MitoQ, AntiOxCIN4, Mito-Esc, and SkQ1 are representative stable mitochondria-targeted conjugates.

MitoQ has the most extensive evidence among these structurally optimized compounds. It consists of a ubiquinone antioxidant moiety linked to TPP through a ten-carbon alkyl chain, enabling its accumulation near the inner mitochondrial membrane and participation in redox cycling [155]. In ATM^+/−^/ApoE^−/−^ mice and high-fat diet-fed mice, MitoQ attenuated hyperglycemia, hyperlipidemia, hepatic steatosis, and mitochondrial oxidative injury and improved body weight- and liver function-related outcomes [156]. A 2024 human mechanistic study using lipid infusion combined with a hyperinsulinemic-euglycemic clamp further demonstrated that MitoQ reduced mitochondrial oxidative stress in skeletal muscle and enhanced insulin-stimulated GLUT4 translocation and glucose uptake [157].

AntiOxCIN4 consists of a dietary caffeic acid-derived antioxidant scaffold linked to TPP through a six-carbon spacer. Structure–activity studies have shown that AntiOxCIN4 exhibits greater mitochondrial targeting and antioxidant activity than its non-targeted parent compound [158]. In mice fed a high-fat, high-sucrose Western diet for 16 weeks, AntiOxCIN4 reduced hepatic lipid accumulation and markers of hepatocellular injury, enhanced fatty acid oxidation and oxidative phosphorylation, and strengthened antioxidant defenses through the PGC-1*α*/SIRT3 axis. It also increased lysosomal proteolytic capacity and restored impaired autophagic flux, thereby linking improved energy metabolism and antioxidant defense with cellular quality control [159,160].

Mito-Esc consists of the natural coumarin esculetin conjugated to TPP through an eight-carbon linker. Early studies demonstrated that Mito-Esc improved endothelial mitochondrial respiration and biogenesis through AMPK/eNOS/SIRT3 signaling and attenuated atherosclerosis in ApoE^−/−^ mice [161]. Subsequent studies in high-fat diet-induced NAFLD/MASH models showed that Mito-Esc reduced hepatic lipid accumulation, inflammation, and fibrosis through the AMPK/SIRT1 axis [162]. In db/db mice, it also suppressed hepatic gluconeogenesis and improved IRS/AKT signaling, insulin resistance, and hyperglycemia-associated atherosclerosis [163]. The consistent validation of Mito-Esc across fatty liver disease, T2D, and cardiovascular complication models strengthens its potential as a mitochondria-targeted candidate for metabolic diseases.

SkQ1 consists of plastoquinone conjugated to TPP through a ten-carbon linker. In alloxan-induced experimental diabetes, preventive administration of SkQ1 reduced blood glucose levels and modulated mRNA and miRNA expression associated with oxidative metabolism [164]. In genetically diabetic db/db mice, SkQ1 did not significantly alter body weight or hyperglycemia but reduced oxidative stress in wounds and improved angiogenesis and skin repair [165]. These findings suggest that the effects of SkQ1 may differ between primary metabolic phenotypes and diabetic complications. Future studies should therefore distinguish its glucose-lowering, mitochondrial antioxidant, and organ-protective effects.

**Table 2:** Delivery and structural optimization strategies for natural small molecules targeting MQC in metabolic diseases.

Formulation or Strategy	Active Compound	Diseases/Models	Optimization Principle	Mitochondrial/MQC-Related Effects	Reference
Phosphatidylcholine liposomes, hPLipo/Cur 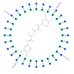	Curcumin	MASH	Improves curcumin solubility, stability, and hepatic exposure	Inhibits NRF2 and promotes nuclear translocation, reduces mtROS, lipid peroxidation, ferroptosis	[146]
Galactose-modified OSL nanoparticles, Gal-OSL/Res 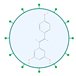	Resveratrol	NAFLD, steatotic HepG2 cells	Enhanced stability and sustained release	AMPK/SIRT1 signaling, FAS/SREBP-1c-mediated lipogenesis, improves insulin resistance	[147]
Galactose-modified PLGA-PEG nanoparticles, EC@PLGA-PEG-GAL NPs 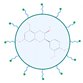	Epicatechin	MASLD, steatotic hepatocytes	Improves hepatocyte uptake, enhances aqueous dispersibility and sustained drug release	AMPK/mTORC1, restores autophagy, increases membrane potential, and reduces mtROS	[148]
Functionalized cationic liposomes for co-delivery, AE@PEP-Lip 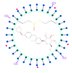	Allicin, empagliflozin	Obesity and differentiated 3T3-L1 adipocytes	Enhanced adipose tissue and mitochondrial exposure	Reduced mtROS and mitochondrial fragmentation, improved respiration, and activated AMPK, UCP1	[149]
Dual-targeted nanoparticles, AST@TPP-WPI-Gal 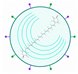	Astaxanthin	NAFLD	Enables dual organ- and organelle-level targeting	Enhances mitochondrial accumulation and antioxidant activity, suppresses lipogenesis	[151]
Mitochondria-targeted polymeric micelles, Cur-CTPP-PEG-PCL 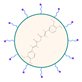	Curcumin	CCl_4_-induced liver fibrosis	Improves encapsulation efficiency, bioavailability, and endosomal escape	Enhances mitochondrial colocalization, MQC endpoints were not systematically evaluated	[152]
TPP-linked mitochondria-targeted conjugate, MitoQ 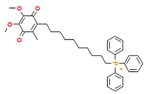	Ubiquinone	Metabolic syndrome, obesity, human insulin resistance	Enabling membrane-potential-driven accumulation	Reduces mitochondrial oxidative stress and enhances GLUT4 translocation and glucose uptake	[155,156,157]
TPP-linked conjugate, AntiOxCIN4 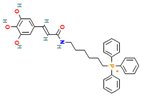	Caffeic acid	NAFLD	Enhance mitochondrial localization and antioxidant activity	Promotes fatty acid oxidation and oxidative phosphorylation, PGC-1α/SIRT3 signaling, and restores autophagic flux	[158,159,160]
TPP-linked conjugate, Mito-Esc 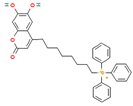	Esculetin	Atherosclerosis, NAFLD/MASH	Facilitate mitochondrial accumulation	AMPK/eNOS/SIRT3, AMPK/SIRT1 signaling, improves mitochondrial respiration and biogenesis, reduces hepatic lipid accumulation, inflammation, and fibrosis	[161,162,163]
TPP-linked antioxidant, SkQ1 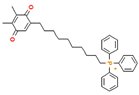	Plastoquinone	Diabetes and diabetic wound models	Promote mitochondrial accumulation	Reduces oxidative stress and improves angiogenesis, with limited effects on hyperglycemia	[164,165]

## 5. Clinical Evidence and Current Clinical-Trial Landscape

Human evidence remains substantially less mature than the cellular and animal literature summarized above. Selected trials have evaluated resveratrol, curcuminoids, astaxanthin, nicotinamide riboside, urolithin A, and MitoQ in obesity, insulin resistance, or fatty liver disease (Table 3). Most studies were small and short in duration and primarily assessed metabolic or imaging-based surrogate outcomes. Direct mitochondrial measurements were uncommon: resveratrol improved skeletal-muscle mitochondrial respiration in obese men [110], whereas nicotinamide riboside did not improve insulin sensitivity or skeletal-muscle mitochondrial respiration, content, or morphology [166,167].

Among interventions with more direct mechanistic relevance, urolithin A produced a mitochondrial molecular signature in older adults [168], and MitoQ reduced mitochondrial oxidative burden and attenuated lipid-induced skeletal-muscle insulin resistance in an acute human study [157]. However, these findings do not establish long-term efficacy in metabolic disease. An ongoing trial is evaluating urolithin A during dietary weight loss in adults with obesity (NCT07377136), including mitochondrial and metabolic biomarkers. No metabolic-disease clinical trials were identified for AntiOxCIN4, Mito-Esc, or SkQ1; their metabolic evidence therefore remains preclinical. Overall, improvements in metabolic surrogates should not be interpreted as proof of MQC engagement unless tissue exposure and mitochondrial endpoints are assessed directly.

## 6. Translational Challenges and Future Perspectives

### 6.1. Limitations of Preclinical Evidence and Causal Validation

The use of natural small molecules to treat metabolic diseases through MQC modulation remains at the stage of mechanistic validation and translational development. Current evidence is derived primarily from cellular and animal studies, which commonly assess ROS production, ATP levels, mitochondrial membrane potential, MQC-related protein expression, and histopathological changes [18,171]. The translational relevance of some preclinical studies is limited by clinically unattainable exposure levels, preventive treatment protocols, and reliance on a single sex or a single disease model. Changes in the expression of PGC-1*α*, PINK1, Parkin, DRP1, or MFN proteins are commonly interpreted as evidence of MQC modulation. Although these measurements alone cannot establish flux, target engagement, or causal necessity, future studies should combine pharmacological interventions with genetic loss-of-function approaches, longitudinal reporters of mitophagy or mitochondrial dynamics, respiratory flux measurements, and tissue-specific validation to improve the reliability of the mechanistic evidence.

### 6.2. Clinical Validation of MQC Modulation and Trial Design

In clinical studies, improvements in glycemia, lipid profiles, body weight, liver enzymes, and hepatic fat content indicate metabolic benefits but do not demonstrate effective MQC modulation in humans without corresponding mitochondrial and mechanistic measurements. Mitochondrial function in peripheral blood mononuclear cells, circulating mtDNA, oxidative stress and inflammatory biomarkers, metabolomic and lipidomic profiles, and imaging-based tissue changes may therefore provide important links between clinical phenotypes and mitochondrial mechanisms [95,168,172]. Clinically meaningful outcomes may include insulin sensitivity assessed by the hyperinsulinemic-euglycemic clamp, hepatic fat content quantified using magnetic resonance imaging proton density fat fraction (MRI-PDFF), histological activity or fibrosis in MASH, physical function, and validated organ-specific outcome measures. Most existing human studies are limited by small sample sizes and short intervention periods and are powered primarily to detect changes in surrogate metabolic outcomes. Clinical trials targeting MQC should therefore prespecify at least one mechanistic endpoint and one clinically meaningful endpoint.

### 6.3. Drug Exposure, Formulation Standardization, and Emerging Technologies

Exposure in vivo is a major determinant of whether mechanisms identified in vitro can be translated into consistent therapeutic effects. Gastrointestinal absorption, gut microbial metabolism, hepatic first-pass metabolism, and tissue distribution substantially influence the actual exposure to active compounds and their metabolites [173,174,175]. Mitochondria-targeted formulations present additional challenges, including membrane potential-dependent accumulation, cation-associated toxicity, inefficient intracellular trafficking, carrier accumulation, and immune responses. Because obesity, T2D, and MASLD/MASH generally require long-term treatment, pharmacokinetics, drug concentrations in target tissues and mitochondria, repeated-dose safety, and drug interactions should be evaluated alongside therapeutic efficacy. Network pharmacology, artificial intelligence, and multi-omics technologies provide valuable tools for characterizing the multitarget effects of natural small molecules [176]. Molecular docking, transcriptomics, proteomics, metabolomics, lipidomics, and single-cell and spatial omics can facilitate target discovery, identify responsive cell populations, and reconstruct regulatory networks [177,178]. However, computational predictions and correlative molecular changes primarily generate mechanistic hypotheses and cannot replace causal validation.

Mitochondrial respiration, ATP production, and membrane potential can be used to assess bioenergetic function, whereas mitochondrial morphology, dynamics, and mitophagic flux can reveal network remodeling and damage clearance. Measurements of mtROS, mtDNA damage, the UPR^mt^, and lysosomal function can further characterize stress adaptation and quality control capacity. Integrating these endpoints with metabolic flux analysis, pharmacokinetics, tissue distribution, and intramitochondrial drug exposure within the same experimental design would clarify whether formulation optimization or structural modification genuinely enhances MQC modulation [179,180,181]. Clinical studies should likewise incorporate reproducible and accessible mechanistic endpoints to establish associations between mitochondrial functional changes, imaging findings, metabolic outcomes, and clinical benefits.

## 7. Conclusions

Mitochondrial quality control dysregulation provides an integrative framework linking nutrient overload, oxidative stress, organelle damage, chronic inflammation, and metabolic reprogramming. Obesity, T2D, and MASLD/MASH are commonly associated with impaired mitochondrial biogenesis, imbalanced fusion and fission, defective mitophagy, insufficient UPR^mt^ adaptation, and increased mtROS- and mtDNA-mediated inflammatory signaling [8,12]. MQC should therefore be viewed as a dynamic network connecting cellular energy homeostasis with metabolic function.

Natural small molecules can simultaneously modulate metabolic signaling, mitochondrial respiration, autophagy, and antioxidant defenses, supporting their use as both pharmacological probes and potential lead compounds. However, current evidence is derived predominantly from cellular and animal studies, and relatively few studies have demonstrated concurrent improvements in mitochondrial function, MQC-related fluxes, and metabolic phenotypes. Poor aqueous solubility, rapid metabolism, and insufficient target-tissue exposure further limit their in vivo efficacy. Liposomes, nanoparticles, polymeric micelles, TPP conjugation, and stimuli-responsive prodrugs can improve pharmacokinetics and mitochondrial delivery but may also introduce risks such as carrier accumulation, immune responses, and mitochondrial toxicity [171,182]. Future studies should therefore integrate pharmacokinetics, tissue and mitochondrial exposure, MQC function, metabolic outcomes, and long-term safety, supported by validation across multiple models and clinically relevant mitochondrial biomarkers.

Overall, natural small molecules targeting MQC for metabolic diseases are at a critical transition from mechanistic discovery to therapeutic translation. Their development into clinically valuable drug candidates will require quantitative confirmation of mitochondrial exposure, causal evidence of MQC modulation, reproducible metabolic benefits across models, and validated long-term safety. Establishing a verifiable, quantifiable, and translational research framework centered on MQC will facilitate the development of natural small molecules for precise mitochondrial intervention and metabolic disease treatment.

## Figures and Tables

**Table 1:** Natural small molecules modulating MQC in metabolic diseases.

Compound	Source	Dose and Duration	Diseases/Models	MQC Process	Pathway/Targets	References
Luteolin 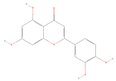	Flavone	0.005% *w*/*w* in HFD orally for 16 w in mice	Fatty liver	Promotes mitochondrial biogenesis and oxidative metabolism	AMPK/PGC-1*α*	[105]
Astaxanthin 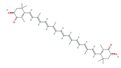	Terpenes	0.02% *w*/*w* in HFD orally for 24 w in mice; 1–50 μM for 24 h in C2C12 cells	Skeletal muscle insulin resistance	Enhances mitochondrial biogenesis, remodeling and oxidative metabolism	AMPK-related signaling	[106]
Melatonin 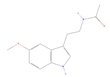	Endogenous indoleamine	20 mg/kg/day orally for 8 w in diabetic mice; 10 mg/kg/day orally for 12 w in ZDF rats; 500 μg/kg/day by i.p. for 4 w in ob/ob mice	Obesity or diabetes models, high-fructose-induced renal injury	Promotes mitochondrial biogenesis, mitophagy, and fatty acid oxidation	CaMKII/AMPK/PGC-1*α*, mitophagy-related signaling	[23,107,108]
Fucoxanthin 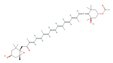	Xanthophyll carotenoid	0.05% *w*/*w* in HFD orally for 40 w in ob/ob mice; 0.1 or 0.25 μM for 48 h in C2C12 cells	Skeletal muscle lipid accumulation, insulin resistance	Enhances mitochondrial biogenesis and fatty acid *β*-oxidation	PKM1, PGC-1*α*/TFAM, PPAR*α*/CPT1	[109]
Resveratrol 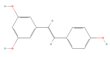	Stilbene polyphenol	150 mg/day orally for 30 d in adults with obesity	Short-term human supplementation studies	Improves mitochondrial energy metabolism and metabolic adaptation	AMPK/SIRT1/PGC-1*α*	[110]
Ginsenoside F2 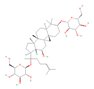	Dammarane-type triterpenoid saponin	50 or 100 mg/kg orally for 4 w in obese mice; 12.5–100μM in 3T3-L1 cells	Adipogenesis and obesity-related models	Suppresses adipogenesis and promotes mitochondrial biogenesis	AMPK	[111]
Ginsenoside Rg3 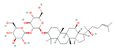	Dammarane-type triterpenoid saponin	2.5 mg/kg by i.p. for 8 w in mice; 40 μM for 24 h in 3T3-L1 cells	Obesity, adipocyte models	Restores thermogenesis and fatty acid oxidation	UCP1 and oxidative metabolism-related signaling	[112,113]
Tanshinone I 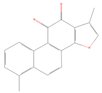	Diterpenoid quinones	2 or 5 mg/kg/day by i.p. for 9 w in mice; 15 μM in 3T3-L1 cells	Obesity, adipocyte models	Promotes mitochondrial biogenesis, fatty acid oxidation, BAT activation, and WAT browning	AMPK and thermogenesis-related signaling	[114]
Tanshinone IIA 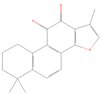	Diterpenoid quinones	6.25–37.5 mg/kg/day orally for 8 w in HFD rabbits; 30 mg/kg orally for 30 d in mice; 10 μM in 3T3-L1 cells	Obesity and diabetes, adipocyte models	Increases mitochondrial content, UCP1-dependent thermogenesis, promotes WAT beiging	AMPK/PGC-1*α*, UCP1	[115,116]
Baicalin 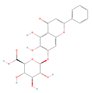	Flavonoid	400 mg/kg/day orally for 12 w in mice; 100 µM for 24 h in HeLa cells or primary hepatocytes	Obesity and hepatic steatosis	Enhances mitochondrial fatty acid uptake and *β*-oxidation	CPT1A	[117]
Alnustone 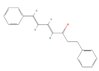	Diarylheptanoid	10 mg/kg/day by i.p. for 5–14 d in mice; 30 mg/kg by gastrogavage for 2 w in mice	MASLD/ MASH	Enhances mitochondrial fatty acid *β*-oxidation	Calmodulin/Ca^2+^ signaling	[118]
Berberine 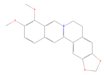	Isoquinoline alkaloid	200 or 300 mg/kg/day by gastrogavage in db/db mice; 100 mg/kg/day by gastrogavage for 8 w in rats	Diabetic nephropathy and insulin resistance models	Restores mitochondrial energy homeostasis and reduces metabolic inflammation	PGC-1*α*, AMPK, Nrf2, NF-κB, NLRP3	[119,133,134]
Epigallocatechin gallate (EGCG) 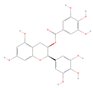	Polyphenol	10–40 μM for 48 h in MIN6 cells	Pancreatic *β*-cell dysfunction	Inhibits excessive fission and mitochondria-mediated apoptosis	DRP1-related apoptotic signaling	[120]
Astragaloside IV 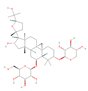	Triterpenoid saponin	100 mg/L for 48 h in hEPCs	Diabetes, high-glucose-treated endothelial progenitor cells	Restores mitochondrial dynamics and reduces mtROS	GSK-3*β*/Nrf2; DRP1, MFN1/2, OPA1	[121]
Coenzyme Q10 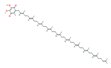	Endogenous lipophilic quinone	20 or 40 mg/kg/day orally for 12 w in HFD-fed rats	HFD models and related clinical studies	Supports electron transport and improves redox balance and mitochondrial dynamics	Electron transport chain and dynamics-related signaling	[122]
Quercetin 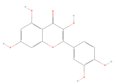	Flavonol	20 mg/kg/day orally for 4 w in mice; 10 or 50 μM for 12 or 24 h in AML12 cells	NAFLD and lipophagy-related models	Activates mitophagy and promotes lipid mobilization and *β*-oxidation	AMPK-mediated mitophagy, lipophagy	[21,123]
Hesperetin 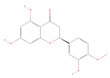	Flavanone	20 or 40 μM for 4 h in HepG2 cells	Palmitate-treated HepG2 cells	Restores membrane potential, ATP production, and mitophagy	PINK1/Parkin; NLRP3	[124]
Lycopene 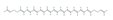	Tetraterpene	10 or 40 mg/kg/day by oral gavage for 12 w in mice; 20 μM for 24 h in AML12 cells	MASLD/ palmitate-treated AML12 cells	Rebalances mitochondrial dynamics, enhances mitophagy, restores mitochondrial function, and reduces apoptosis	DRP1, MFN2/OPA1, PINK1/Parkin-mediated mitophagy	[125]
Myricetin 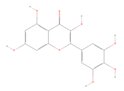	Flavonol	25–100 mg/kg/day orally for 8 w in mice	MASLD/MASH	Enhances mitophagy, mitochondrial function, and fatty acid *β*-oxidation	PINK1 stabilization and PINK1/Parkin-dependent mitophagy	[126]
Kaempferol 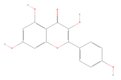	Flavonol	50–200 mg/kg/day orally for 12 w in DKD rats; 50 μM for 24 h in HK-2 cells	Diabetic nephropathy and AGEs-treated HK-2 cells	Restores mitochondrial fusion, mitophagy, autophagic flux, and mitochondrial function	MFN1/OPA1, DRP1/FIS1, PINK1/Parkin-mediated mitophagy	[127]
Diosgenin 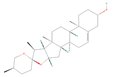	Natural steroidal sapogenin	10 or 20 mg/kg/day orally for 8 w in DN rats; 1–4 μM for 24 h in HK-2 cells	Diabetic nephropathy and high-glucose-treated HK-2 cells	Restores autophagic flux and mitophagy, suppresses mitochondrial fission and promotes fusion	CaMKK2/AMPK/mTOR, PINK1/MFN2/Parkin, DRP1, MFN1/2/OPA1	[128]
Curcumin 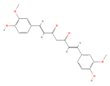	Polyphenol	2% *w*/*w* in HFD orally for 10 w in rats; 1% or 3% *w*/*w* for 21 w in ob/ob mice	Obesity and Diabetes	Improves redox homeostasis, mitochondrial biogenesis, complex I activity, and ATP production	SIRT3, NF-*κ*B, NRF1, TFAM, complex I, GSH	[20,130]
Nobiletin 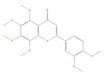	Flavonoid	50 mg/kg/day by i.p. for 4 w in BALB/c mice; 50 or 100 μM for 24 h in AML12 cells	NASH and palmitate-treated AML12 cells	Restores complex I activity, ATP production, and mitochondrial Ca^2+^ homeostasis	Complex I and mitochondrial Ca^2+^ uptake-related signaling	[22]
Matrine 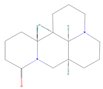	Quinolizidine alkaloid	0.5, 2.5, or 10 mg/kg for 8 w in HFD mice; 0.5, 2.5, or 10 mg/kg for 4 w in MCD mice; 200–800 μM for 12 h in L02 cells.	NAFLD/NASH, palmitate-treated L02 cells	Restores ER–mitochondrial Ca^2+^ homeostasis and mitochondrial membrane potential, reduces oxidative stress and apoptosis	SERCA-dependent Ca^2+^ regulation, PERK/IRE1/ATF6 signaling	[131]
Nicotinamide riboside 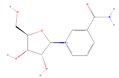	Pyridine nucleosides	400 mg/kg/day orally for 18 w (prevention) or 9 w (therapy) in mice; 500 mg/kg/day for 7 w in ApoE^−/−^ mice;1 mM for 24 h in primary hepatocytes.	NAFLD, primary hepatocyte	Replenishes NAD^+^ and induces an adaptive UPR^mt^, enhancing mitochondrial protein folding, degradation, and respiratory chain assembly	SIRT1/SIRT3, CLPP and HSP10	[137]
Agnuside 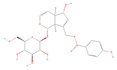	Iridoid glycoside	10 mg/kg/day by i.p. for 4 w in mice; 7.5 mg/kg/day by i.p. for 8 or 12 w in mice	HFD-induced obesity and insulin resistance	Stabilizes mitochondrial proteostasis and respiratory chain assembly	NDUFAF6 stabilization and reduced ubiquitination	[138]

**Table 3:** Selected completed and ongoing clinical studies relevant to metabolic disease.

Intervention/Status	Design	Regimen	Main Finding	Reference
Resveratrol, Completed	Obese men; randomized double-blind crossover	150 mg/day, oral; 30 days	Improved selected metabolic measures and reduced intrahepatic lipid content	[110]
Nicotinamide riboside, Completed	Obese insulin-resistant men; randomized placebo-controlled	1000 mg twice/day, oral; 12 weeks	No improvement in insulin sensitivity or whole-body glucose metabolism	[166,167]
Urolithin A, Completed	Healthy sedentary older adults; first-in-human study	500 or 1000 mg/day, oral; 4 weeks	Favourable short-term safety and modulation of plasma acylcarnitines	[168]
MitoQ, Completed	Men with overweight/insulin resistance; randomized crossover lipid-infusion study	Single 160 mg oral exposure; acute	Attenuated lipid-induced skeletal-muscle insulin resistance	[157]
Urolithin A, Ongoing	Adults with obesity undergoing dietary weight loss; randomized placebo-controlled	500 mg/day, oral; 24 weeks	Weight loss, functional muscle preservation, and cardiometabolic outcomes under evaluation	NCT07377136
Curcuminoids + piperine, Completed	NAFLD; randomized double-blind placebo-controlled	500 mg + 5 mg/day, oral; 8 weeks	Improved inflammatory markers and ultrasound-assessed steatosis	[169]
Astaxanthin, Completed	Prediabetes with dyslipidaemia; randomized placebo-controlled	12 mg/day, oral; 24 weeks	Reduced LDL cholesterol and selected cardiovascular-risk markers; insulin-action endpoint was not significant	[170]

## Data Availability

No new data were created or analyzed in this study. Data sharing is not applicable.

## Abbreviations

MQC, mitochondrial quality control; T2D, type 2 diabetes; MASLD, metabolic dysfunction-associated steatotic liver disease; MASH, metabolic dysfunction-associated steatohepatitis; ROS, reactive oxygen species; mtROS, mitochondrial ROS; mtDNA, mitochondrial DNA; AMPK, AMP-activated protein kinase; SIRT1, NAD^+^-dependent deacetylase sirtuin; PGC-1*α*, peroxisome proliferator-activated receptor gamma coactivator 1-alpha; NRF1, nuclear respiratory factor 1; GABP, GA-binding protein; TFAM, mitochondrial transcription factor A; MFN1, mitofusin 1; MFN2, mitofusin 2; OPA1, optic atrophy 1; DRP1, dynamin-related protein 1; FIS1, mitochondrial fission 1 protein; PINK1, PTEN-induced kinase 1; LC3, microtubule-associated protein 1 light chain 3; PHB2, prohibitin 2; AMBRA1, autophagy and beclin 1 regulator 1; UPR^mt^, mitochondrial unfolded protein response; HSP, heat shock protein; CLPP, caseinolytic peptidase P; ATF5, activating transcription factor 5; CHOP, C/EBP homologous protein; cGAS–STING, cyclic GMP-AMP synthase-stimulator of interferon genes; TLR9, toll-like receptor 9; NLRP3, NLR family pyrin domain-containing 3; UCP1, uncoupling protein 1; GLUT4, glucose transporter type 4; TPP^+^, triphenylphosphonium.
